# LED and HPS Supplementary Light Differentially Affect Gas Exchange in Tomato Leaves

**DOI:** 10.3390/plants10040810

**Published:** 2021-04-20

**Authors:** Onofrio Davide Palmitessa, Aina E. Prinzenberg, Elias Kaiser, Ep Heuvelink

**Affiliations:** 1 Department of Agricultural and Environmental Science, University of Bari Aldo Moro, 70126 Bari, Italy; 2 Horticulture and Product Physiology, Wageningen University and Research, PO Box 16, 6700 AA Wageningen, The Netherlands; aina.prinzenberg@gmx.de (A.E.P.); elias.kaiser@wur.nl (E.K.); ep.heuvelink@wur.nl (E.H.); 3 Plant Breeding Laboratory, Wageningen University and Research, PO Box 386, 6700 AJ Wageningen, The Netherlands

**Keywords:** greenhouse, photosynthesis, stomatal conductance, thermal imaging, transpiration rate, leaf temperature

## Abstract

Using light emitting diodes (LED) instead of conventionally used high pressure sodium (HPS) lamps as a supplemental light source in greenhouses results in a higher efficacy (µmol light per J electricity) and makes it possible to customize the light spectrum. To explore the effects of LED and HPS on gas exchange, thermal relations, photosynthesis, and water status of young tomato plants, seven genotypes were grown in a greenhouse under LED (95% red, 5% blue) or HPS lamps in four experiments differing in the fraction of lamp light over natural light. HPS lights emit a broader spectrum of red (40%), green–yellow (50%), blue (5%), and far-red (5%) and a substantial amount of infrared radiation (heat). Young tomato plants grown under LED showed lower leaf temperature and higher stomatal density, stomatal conductance (*g_s_*) and transpiration rate (*E*) than plants grown under HPS; this may be due to the different supplemental light spectrum. The young plants grown under LED tended to have increased photosynthetic capacity. Furthermore, the water stress indices CWSI and I_G_, which were obtained using thermal imaging, were positively correlated with gas exchange-derived *g_s_* and *E*, putting forward the use of thermal imaging for the phenotyping of transpiration. Under LED light, photosynthetic gas exchange was generally increased, which agreed with the water stress indices. The extent of this increase was genotype-dependent. All differences between LED and HPS were smaller in the experiments where the fraction of lamp light over natural light was smaller.

## 1. Introduction

In the northern hemisphere, tomato nurseries must meet the peak demand for transplants during a period when the daily light integral (DLI; mol photons m^−2^ s^−1^) of natural light is lowest [1]. Supplementary light (SL) is frequently applied to improve tomato seedling quality [2,3,4,5], and high-pressure sodium lamps (HPS; [6]) are commonly used as a supplementary light source in greenhouses. HPS emit photosynthetically active radiation (PAR), but also infrared radiation [7], heating up the leaves. More recently, light emitting diodes (LED) have been investigated as a possible alternative for HPS in greenhouses. Their efficacy (µmol per J) is higher compared to HPS lamps [8], with less radiant heat output and a longer lifetime compared to HPS. LED and HPS lamps dissipate thermal energy differently. While LED fixtures dissipate a large part of heat through natural or forced convection, HPS lamps have a high operating temperature and emit longwave radiation (3000–100,000 nm) in the same direction as photosynthetically active radiation, i.e., towards the plants [9]. LED also allows one to customize the light spectrum. However, to optimize light use efficiency, plants must adapt their physiology and morphology to a given light spectrum in a process called light acclimation [10]. Acclimation may also include changes in photosynthetic capacity, which can be brought about by changes in the concentration of, e.g., Rubisco [11]. Acclimation of a plant to a light environment is characterized by changes in its phenotype (environmental plasticity) and is influenced by the plant’s genetics [12]. For instance, Liu et al. [13] and Wang et al. [14] found that during long-term acclimation to specific light spectra, stomatal morphology, density and opening rates were changed, with effects on overall gas exchange. Apart from the light spectrum, plants will likely need to adapt differentially to the reduced heat radiation when grown under LED compared to HPS light. For example, Nelson and Bugbee [9] found that LED modules reduced leaf temperature compared to HPS lamps. Giuliani et al. [15] found that leaf temperature affects leaf anatomy during development, biochemistry (including photosynthetic capacity), transpiration and stomatal conductance. Stomatal conductance is an important physiological trait, as stomatal behavior impacts photosynthetic CO_2_ uptake, transpiration, and temperature, all of which influence plant growth and water status [16]. The light spectrum influences the regulation of stomatal movements, and the combination of red (R) and blue (B) light stimulates stomatal opening [17,18]. Eisinger et al. [19] found that sole green (G) light also induced stomatal opening. However, G light reversed the B light-stimulated opening of stomata when it was given simultaneously with continuous B-light [20,21]. It is argued that the B:G light ratio is a signal for the plants to regulate stomatal conductance, in order to balance photosynthesis and water use efficiency [22]. Leaf gas exchange is further affected by the genotype [23]. Therefore, the impact of the supplemental light spectrum on transpiration can be expected to be genotype-dependent. Diversity between varieties provides the opportunity to discover genotypes with desirable traits (e.g., high-water use efficiency) that, when crossed with high-yielding varieties, could produce progenies with improved performance and yield under a given environment. However, identifying individuals with the desired eco-physiological and agronomic responses and traits requires the development of appropriate phenotyping tools [24]. In this regard, thermography can be used to derive estimates of stomatal conductance under a dynamic environment, thereby opening up a new avenue for plant phenotyping and selection [25]. From thermographic images, different parameters can be obtained: the thermal index (I_G_), which describes thermal differences and the crop water stress index (CWSI), which is an index for water stress—both are positively correlated with stomatal conductance [26,27].

LED can improve the biomass production of young tomato plants due to the higher photosynthetic efficiency of the plants, compared to HPS lamps [5], but the underlying physiological process(es) are not well understood. During this study, we hypothesize that leaves of young tomato plants grown under HPS have higher temperature and therefore higher evaporative demand than leaves grown under LED. Therefore, leaves grown under HPS might have higher stomatal conductance, transpiration rate and stomatal density; furthermore, we hypothesize that the extent of acclimation to a given light source is genotype-dependent. At the same time, G radiation in HPS lamps may reverse B light-induced stomatal opening, thus possibly reducing gas exchange and photosynthetic efficiency. The aim of this study was to determine how two different supplemental lights, HPS and LED, influence gas exchange parameters of different young tomato plants, with a focus on leaf temperature. Thermography was assessed as a technique to determine differences between genotypes in stomatal conductance and transpiration rate from CWSI and I_G_.

## 2. Results

### 2.1. Stomatal Conductance (g_s_) and Transpiration Rate (E)

**Figure 1:**
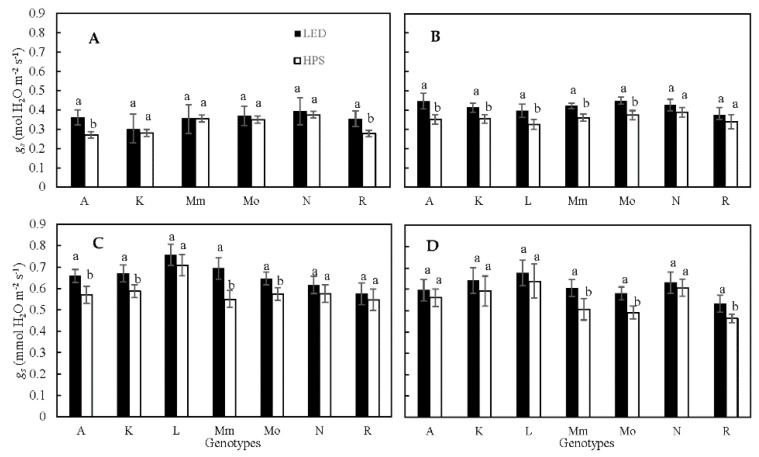
Stomatal conductance (*g_s_*) per genotype grown under HPS and LED supplemental light (measured using porometer), during Expt. 1–4 (**A**–**D**). Values are the average of measurements from 3 plants per genotype. Different letters above bars within a panel and for each genotype separately indicate significant differences according to Fisher’s protected LSD test (*p* = 0.05). Error bars show ± SD. A—Ailsa Craig; K—Kentucky Beefsteak; L—LA1578; Mm—Moneymaker; Mo—Momotaro; N—Nunhems-FM00; R—Rutgers.

Based on porometer measurements, plants grown under LED showed a significantly higher *g_s_* (*p* < 0.05 or *p* < 0.001, depending on the experiment) than plants grown under HPS lamps; on average, *g_s_* was 6% higher under LED (Table 1). Across experiments, ‘Rutgers’ showed the lowest *g_s_* and ‘LA1578’ showed the highest *g_s_* values. Additionally, there was a significant genotype by light treatment interaction (Table 1) that varied per experiment (Table 1, Figure 1). The ratio of *g_s_* under LED divided by *g_s_* under HPS across experiments showed no significant differences between genotypes (Table S1). However, while most genotypes showed an on average between 4 and 20% higher *g_s_* under LED light, the genotype ‘LA1578’ had a 5% lower *g_s_* under LED than under HPS light (Figure 1). Thus, lamp type effects on *g_s_* were genotype-specific.

The highest values for transpiration (*E*), measured using the LI-6400, were again found in plants grown under LED (2–24% higher than HPS depending on experiment; Table S1). Similarly, as for *g_s_*, ‘Rutgers’ showed low values for *E* across treatments in all experiments, but these were not significantly different from those of other genotypes (Table S2).

### 2.2. Stomatal Density

Leaves grown under LED showed an on average ~14% higher stomatal density compared to HPS (Table 2), concomitant with an increase in *g_s_* under LED. The difference in stomatal number per unit area in LED compared to HPS varied between genotypes, with significant differences of ca. 11%, 18% and 37% for the genotypes ‘Moneymaker’, ‘Nunhems’ and ‘Rutgers’, respectively (Table 2). Across experiments 3 and 4, the lowest stomatal density was found among the genotypes ‘Rutgers’ and ‘Ailsa Craig’ (Table 2). The genotype with the highest stomatal density was ‘LA1578’, again matching with the highest *g_s_* for this genotype (Table 1). The difference in stomatal density between ‘Rutgers’ (Figure S1) and the genotype ‘LA1578’ was significant under both light conditions. ‘LA1578’ had 49% and 57% more stomata than ‘Rutgers’ under HPS and LED light, respectively (Table 2).

### 2.3. Leaf Temperature

Leaf temperature tended to be higher under HPS compared to LED lamps, although large differences between single experiments were apparent (Table 3). Variation between experiments was most likely caused by differences in sunlight intensity (Figure S2). In the first two experiments, a significantly higher leaf temperature was measured under HPS: this difference was 1.8 °C in experiment 1 (*p* < 0.001), and 0.8 °C in experiment 2 (*p* < 0.001). No leaf temperature differences were found in the two consecutive experiments (Table 3), where sunlight represented a larger part of total PPFD (Figure S2). No differences in average leaf temperature were found between genotypes.

### 2.4. Plant Water Stress Indices

**Figure 2:**
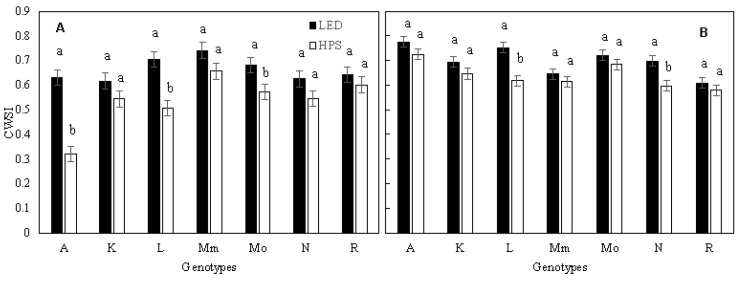
Crop water stress index (CWSI) per genotype, grown under HPS and LED supplemental light. Values are the average of 3 plants per genotype in experiment 2 (**A**) and experiment 3 (**B**). Different letters above bars within a panel and for each genotype separately indicate significant differences according to Fisher’s protected LSD test (*p* = 0.05). Error bars show ± SD. A—Ailsa Craig; K—Kentucky Beefsteak; L—LA1578; Mm—Moneymaker; Mo—Momotaro; N—Nunhems-FM001; R—Rutgers.

Thermography was used as a technique to detect possible differences in physiologically desirable traits between the genotypes and to estimate *g_s_* and *E* from CWSI and I_G_. In the first three experiments, CWSI was significantly (*p* < 0.01) lower, by 5–18%, in plants grown under HPS than under LED light (Table 4); during experiment 2, the largest treatment effect was seen for LA1578, with a 24% reduced CWSI in HPS compared to LED. The smallest impact of light treatments was found in ‘Rutgers’, with a decrease of only 2% (Figure 2). Like the CWSI, I_G_ was increased by between 14 and 32% under LED compared to HPS during the first three experiments, while in the fourth experiment no difference could be identified (Table S3).

### 2.5. CO_2_ Response of Net Photosynthesis Rate

**Figure 3:**
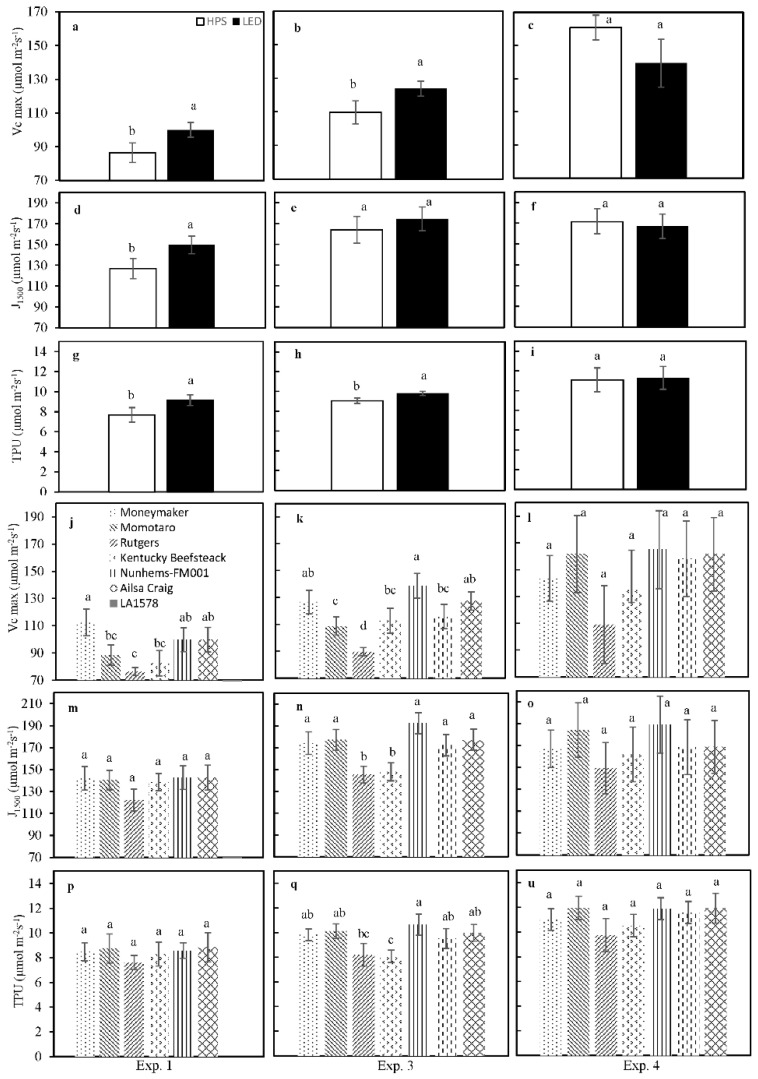
Photosynthetic capacity as affected by supplemental lighting type (HPS or LED) and genotype. V_c,max_, J_1500_ and TPU compared in experiment (Exp.) 1 (**a**,**d**,**g**,**j**,**m**,**p**), Exp. 3 (**b**,**e**,**h**,**k**,**n**,**q**) and Exp. 4 (**c**,**f**,**i**,**l**,**o**,**u**) between the supplemental light conditions (from **a** to **i**) and between genotypes (from **j** to **u**). Different letters on top of a bar within each panel indicate significant differences according to Fisher’s protected LSD test (*p =* 0.05). Error bars show ± SE (supplementary light: *n* = 21; genotypes: *n* = 3).

To assess the effects of SL type on photosynthetic capacity, the response of net photosynthesis rate to internal CO_2_ partial pressure was measured in experiments 1, 3 and 4, and the parameters V_c,max_, J_1500_ and TPU were extracted by curve fitting (Figure 3). In experiments 1 and 3, all three parameters were significantly higher in leaves grown under LED compared to those under HPS (Figure 3); there were no differences between light treatments in experiment 4. V_c,max_ was the parameter that differed most strongly between genotypes, with ‘Rutgers’ and ‘Kentucky Beefsteak’ showing significantly lower values during experiments 1 and 3 (Figure 3J,K). J_1500_ and TPU were less variable between genotypes, as both were only significantly reduced compared to the other genotypes in ‘Rutgers’ and ‘Kentucky Beefsteak’ in experiment 3 (Figure 3E,F).

### 2.6. Correlations between Physiological Parameters

**Figure 4:**
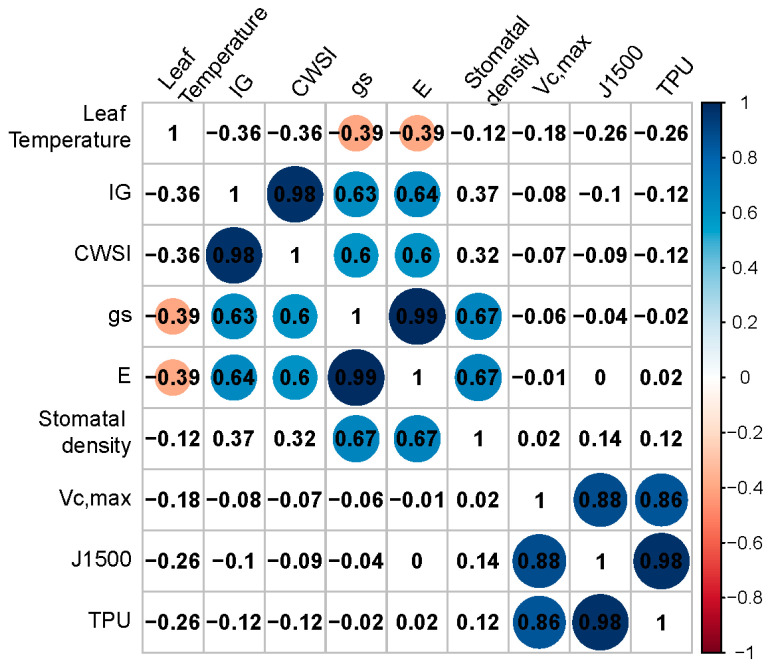
Correlation matrix between leaf temperature, thermal index (I_G_), crop water stress index (CWSI), stomatal conductance (*g_s_*), transpiration rate (*E*), stomatal density, maximum carboxylation rate (V_cmax_), electron transport rate at 1500 μmol m^−2^ s^−1^ (J_1500_) and maximum triose phosphate utilization rate (TPU), across all experiments (4 experiments × 7 genotypes, hence *n* = 28). Numbers represent Pearson’s correlation coefficient; blue circles represent positive correlations (*p* < 0.05) and red circles represent negative correlations (*p* < 0.05). Size of circle depends on height of correlation, while in the cells without circle, no statistically significant correlations were found. Pearson’s correlation test was performed.

Using individual data across SL conditions and genotypes, a positive correlation was found between *g_s_* and stomatal density (r = 0.67; Figure 4) and between *E* and stomatal density (r = 0.67; Figure 4). *g_s_* and *E* were positively correlated with CWSI and I_G_ (r = 0.58–0.72; Figure 4). Leaf temperature and *E* are the variables used to calculate g_s_ in the LI-6400, hence (strong) correlations between these variables are to be expected. Between *g_s_* and E, the correlation coefficient was 0.99, while between CWSI and Ig, this was 0.98 (Figure 4). Significant (*p* < 0.05) negative correlations were found between leaf temperature and *g_s_* and E (r = −0.39; Figure 4), in agreement with the cooling capacity of leaf transpiration. Significant positive correlations were found among parameters related to photosynthetic capacity (V_c,max_, J_1500_ and TPU), while no correlations were found between these and other physiological parameters (Figure 4).

## 3. Discussion

Seven tomato genotypes were grown under HPS or LED supplementary light in four experiments, differing in the ratio between SL and natural light. The aim of this study was to determine how HPS and LED influence gas exchange parameters of different tomato genotypes, with a focus on leaf temperature. The experiments show reproducible differences between the compartments with the different SL. 

### 3.1. High Pressure Sodium (HPS) and LED Supplemental Light Influenced Leaf Temperature

The plants under HPS light are more strongly exposed to heat radiation from the lamps and Nelson and Bugbee [9] reported that about 95% of all longwave radiation is absorbed by leaves, increasing leaf temperature. According to Nelson and Bugbee [9], we observed higher leaf and meristem temperatures under HPS than under LED (Table 3). This was likely due to a larger fraction of longwave and IR radiation from HPS than LED (Table 5) and a lower rate of transpiration (Table 1 and Table S2). Differences in leaf temperature between HPS and LED were smaller when the fraction of SL in total light decreased (Experiment 4; Figure S2). Since transpiration was not higher in experiment 4, similar leaf temperatures for HPS and LED may be due to the IR provided by the sun that warmed up the leaves of plants under LED. This would support the idea that the plants did not adapt their rate of transpiration with increasing IR and leaf temperature was primarily controlled by irradiation. Transpiration capacity decreased under HPS because of lower *g_s_* compared to LED. On the interaction between leaf temperature and stomatal behavior, the literature is often contradictory: Mott and Peak [28] found that with increasing leaf temperature, *g_s_* increased; Von Caemmerer and Evans [29] observed that some species showed large increases in *g_s_* with increasing leaf temperature; Lahr et al. [30] found that increasing leaf temperature stimulated stomatal closure; Bañon et al. [31] observed a negative correlation between leaf temperature and stomatal density; and Farquhar and Sharkey [32] observed negative correlations between *g_s_* and leaf temperature. These contradictory results are probably due to several other factors that can affect both leaf temperature and stomatal behaviour (environmental conditions, genotypes, cultivation technique, etc.). While leaf temperature was negatively correlated with *g_s_* and *E*, stomatal density was positively correlated (*p* ≤ 0.001) with both (Figure 4), according to the findings of Zhenzhu and Guangsheng [33]. Stomatal conductance is a measure of the degree of stomatal opening and density and is a physiological variable related to the regulation of water and carbon assimilation. Moreover, *g_s_* can be used as an indicator of plant water status [34]. Transpiration rate (*E)* is closely related to water movement through a plant and its transpiration from leaves, stems, and flowers. *E* is integral to the calculation of *g_s_*, as is leaf temperature. Leaves from plants grown under HPS showed lower *g_s_* and *E* than from plants grown under LEDs (Figure 1). Therefore, since the large genotypic effects found for *g_s_*, *E* and stomatal density were not found back in the leaf temperature difference of the genotypes, there seem to be other physiological factors, such as stomata opening or leaf architecture, linked to leaf temperature regulation. In fact, if leaf temperature did not differ much between genotypes, ‘Ailsa Craig’ and ‘Rutgers’ showed the lowest *g_s_* and stomatal density and ‘LA1578’, on average, showed the highest *g_s_* and stomatal density (Table 1 and Table 2). This confirms the hypothesis that tomato plant gas exchange varied, responding to the supplemental light spectrum and that acclimation of *g_s_*, *E* and stomatal density is genotype-dependent [12].

### 3.2. The Effects of Supplemental Light Spectral Quality on Plant Physiology

Experiments 1, 2 and 3 were conducted during the fall–winter period, when the natural light photoperiod was, respectively, 9, 7 and 9 h per day. This means that plants received only SL, respectively, for 7, 9 and 7 h per day, while during experiment 4 (conducted during winter-spring period), we had 3 h per day with only SL as the light source (Figure S2). Therefore, a greenhouse is not the best place to study the effect of SL spectral quality on the crop, because there is sunlight influence. Anyway, during this research activity, the young tomato plants received a great part of the radiation from SL fixtures (Figure S2), and in particular for the first three experiments, SL was the sole lighting source for a period between 7 and 9 h per day before sunrise. As the plants were grown mainly with SL radiation rather than solar light radiation, we hypothesized that we could find different physiological behaviours in response to different SL spectral qualities. In fact, HPS has a lower fraction of red (600–700 nm) compared to the LED light used in our experiments (Table 5). Furthermore, HPS light contains a substantial fraction of G–Y (500–600 nm), while LED did not (Table 5). The addition of G light to a R/B background has previously been shown to reverse B light-induced stomatal opening [20,21], and this has often been associated with a decrease in *g_s_* and *E* [13,35,36]. While IR wavelength and the thermal energy dissipation of HPS lamps contributed to increase leaf temperature compared with LED, the lower B:G ratio in HPS could be a signal for the plants to preserve their water status, thereby reducing *g_s_* and *E* [22]. According to previous studies [13,22,35,37], we found that the plants grown under the lowest B:G SL ratio (HPS) showed the lowest stomatal density (Table 2). *g_s_* was higher under LED than HPS (Figure 1), except for ‘LA1578’, which showed a higher *g_s_* under HPS. For *E,* the same trend as for *g_s_* was observed (Table S2). Moreover, not only water vapour exchange but also photosynthetic capacity could be influenced by different SL spectra. In fact, specific regions in the shortwave spectrum can have strong effects on leaf light acclimation and, consequently, photosynthetic capacity [38,39,40,41]. In our study, global values of two parameters expressing photosynthetic capacity, maximum carboxylation rate (V_c,max_) and electron transport rate at 1500 μmol m^−2^ s^−1^ PPFD (*J*_1500_) were significantly higher under LED (experiments 1 and 3, not in experiment 4; Figure 2). Although leaves under LED experienced slightly reduced temperatures compared to HPS (Table 3), they may have increased their photosynthetic machinery to correct for differences in enzyme turnover, which is strongly temperature-controlled [42,43]. The fact that the largest differences between HPS and LED in both leaf temperature (Table 3) and photosynthetic capacity (Figure 2) were observed in experiment 1 and experiment 2 underlines this hypothesis, as in these experiments the ratio between SL and solar light was highest. Additionally, given that HPS lamps had a larger output in both the G–Y and the FR region, it is tempting to speculate that the combination of these two colours led to shade acclimation (as discussed in Smith et al. [22]), which among other things would result in reduced photosynthetic capacity in these leaves. Between genotypes, V_c,max_ differed more strongly than J_1500_ and TPU; this suggests that Rubisco concentrations are more strongly affected by genotypic differences than either electron transport or triose phosphate utilization capacity.

### 3.3. Plant Water Stress Indices for Phenotyping of Transpiration

Stomatal conductance is often considered an important trait for future yield improvements, as it is positively related to photosynthesis [16]. Thermography was assessed as a technique to determine differences between genotypes in stomatal conductance and transpiration rate from CWSI and I_G_. We measured *g_s_* with a porometer and a LI6400. Based on the studies of Jones [44] and Idso [45], *g_s_* can be derived from I_G_ and CWSI non-invasively and potentially as high-throughput scalable measurements. CWSI can vary between 0 (no transpiration/ bad water status) and 1 (maximal transpiration/good water status). Young tomato plants grown under supplemental LED compared to HPS showed better water status (higher CWSI values; Table 4) and therefore a higher transpiration capacity. Positive correlations between I_G_, CWSI, *g_s_* and E, as described in previous studies [27,45,46], were confirmed. Our hypothesis was that the climatic conditions under LED and the spectral quality of the LED modules positively influenced stomata behaviour and the gas exchange system, improving the overall plant water status compared with HPS treatment. Moreover, ‘Rutgers’ showed a value of CWSI that was not significantly different between HPS and LED, while for ‘LA1578’, CWSI was lower under HPS than under LED (Figure 3). Therefore, preliminary measurements for each genotype will be necessary (to find the leaf absorption coefficient [47]) to use this technique to predict *g_s_*. With thermography, it is possible to estimate plant water status and to predict *g_s_* using rapid and noninvasive measurements—this may open up possibilities, especially in the field of plant genetic improvement.

## 4. Materials and Methods

### 4.1. Plant Material and Growth Conditions

**Figure 5:**
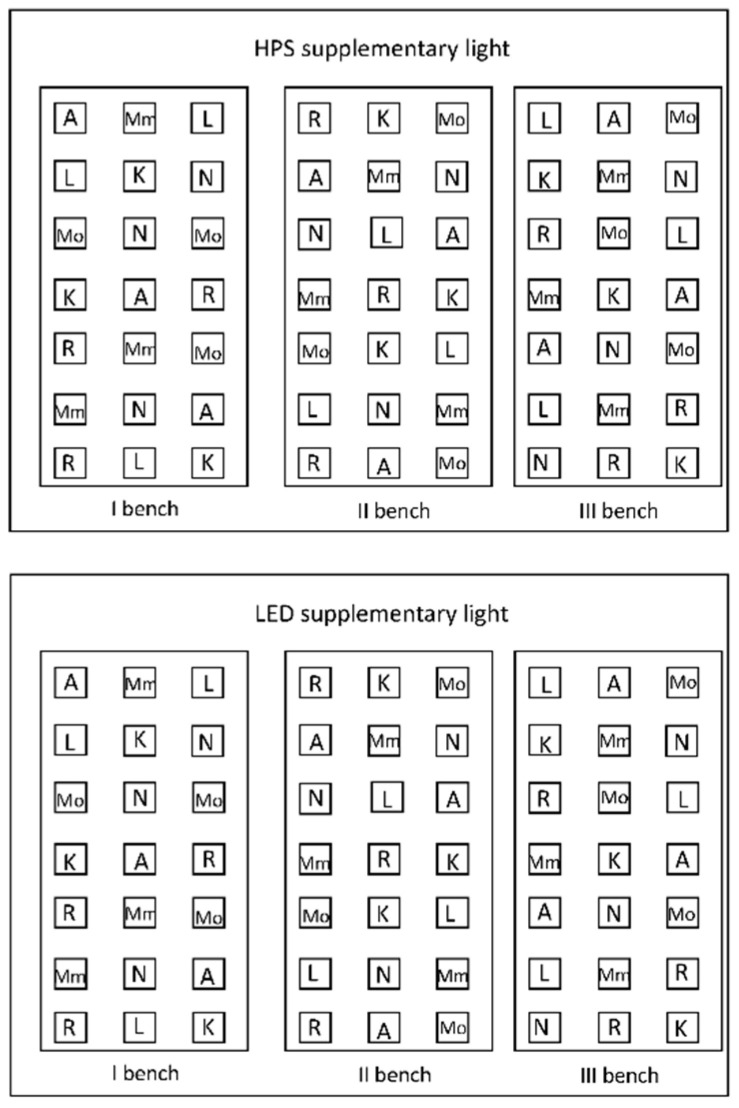
Experimental layout. Tomato plants (7 genotypes) grown in two adjacent greenhouse compartments of each 60 m^2^ with different supplementary light technologies (HPS vs. LED). A–Ailsa Craig; K–Kentucky Beefsteak; L–LA1578; Mm–Moneymaker; Mo–Momotaro; N–Nunhems-FM001; R–Rutgers. The plants were interspaced by other tomato plants (that were not evaluated in this study) growing ca. 8 cm apart.

Seven tomato genotypes (*Solanum lycopersicum* L.: ‘Moneymaker’, ‘Momotaro’, ‘Rutgers’, ‘Ailsa Craig’, ‘Kentuky Beefsteak’ and ‘Nunhems–FM001’; and *Solanum pimpinellifolium* L.: ‘LA1578’) were grown in four greenhouse experiments during the 2018–2019 winter/spring season: experiment 1 (Oct.–Nov.), experiment 2 (Dec.–Jan.), experiment 3 (Jan.–Feb.) and experiment 4 (Mar.–Apr.). Seeds were sown on stonewool plugs (Grodan, Roermond, The Netherlands) and germinated in the glasshouse facilities (Unifarm) of Wageningen University, the Netherlands (53 °N, 5.5 °E), under supplemental HPS or LED light. Seven days after sowing, seedlings in plugs were transferred to 10 × 10 × 6 cm stonewool blocks (Grodan) and placed on ebb and flow benches, for four weeks in experiment 1 and for ca. three weeks in the other experiments (three plants per genotype were placed on each bench), under either HPS and LED red/blue supplemental lighting. The experimental layout consisted of three benches per supplemental lighting condition, with three plants of each genotype on each ebb and flow bench. Nine plants for each genotype were grown under HPS and nine plants under LED (Figure 5). HPS-lighting (Master green power, cgt 400W, Signify, Eindhoven, The Netherlands) or DR/LB LED-lighting (Green Power LED top lighting module, 190W, Signify) were applied at about 200 µmol m^−2^ s^−1^ photosynthetic photon flux density (PPFD) at plant level, and lamps were switched on 16 h before sunset and switched off at sunset. HPS light contains a substantial fraction of yellow (Y) to G (501–600 nm), while LED did not (Table 5). The 501 to 600 nm range of HPS light two peaks in the G–Y light segment at 550 nm and at 580 nm [48]. For LED fixtures, the emission peaks were at ca. 650 and 450 nm, having peaks only in B and R regions with very little output in the FR region (Table 5). 

A standard nutrient solution (pH = ca. 5.5, EC = 2.0 dS m^−1^, NH_4_ = 1.2, K = 7.2, Ca = 4.0, Mg = 1.82, NO_3_ = 12.4, SO_4_ = 3.32, P = 1.0 mmol L^−1^ and Fe = 35.0, Mn = 8.0, Zn = 5.0, B = 20.0, Cu = 0.5, Mo = 0.5 μmol L^−1^) was used for fertigation. Plants were watered daily, without differences between the compartments. Average air temperature during the day from the first to the fourth experiment was, respectively: 22.8, 22.9, 23.2 and 24.8 °C; air temperature during the night was, respectively: 19.2, 19.2, 19.4 and 19.5 °C (Figure S3). Relative air humidity (RH) was set at 70%. When global radiation outside the greenhouse exceeded 200 W m^−2^, a shading screen (42% light reduction, Harmony 4215 O FR) was closed to prevent excessive sunlight from entering the greenhouse. To prevent light pollution from adjacent compartments, all side walls of the greenhouse compartments were closed off, using white horticultural plastic foil. The greenhouse climate was recorded by a horticultural sensor box (Hoogendoorn-Economic; Hoogendoorn, Vlaardingen, the Netherlands). During each experiment, the environmental conditions between the two experimental compartments, apart from SL treatment spectra, were kept identical. For experiment 1, the daily light integral (DLI) received by the plants (combination of natural light and SL) was on average 20 mol m^−2^ d^−1^ and 75% of the total light was SL (Figure S2); the average DLI in experiment 2 was similar to experiment 1 (18.8 mol m^−2^ d^−1^; Figure S2); however, the fraction of SL in total light was higher (82%; Figure S2); for experiment 3, the average DLI was 21.3 mol m^−2^ d^−1^, and 73% of it was supplemental light (Figure S2); finally, during experiment 4, the average DLI was 29 mol m^−2^ d^−1^, and therefore was much higher than in experiments 1–3, while the fraction SL in total light was only 61% (Figure S2).

### 4.2. Daily Light Integral (DLI) and Light Quality

To determine the greenhouse transmissivity, PPFD at plant level was measured on a cloudy day with a quantum sensor (LI-191SA, LI-COR Biosciences, Lincoln, NE, USA) while concomitantly measuring global radiation (W m^−2^) outside the greenhouse using a solarimeter (Kipp en Zonen, Delft, The Netherlands). The fraction of photosynthetic photon flux (PPF; 400–700 nm) in the total global radiation was assumed to be 47% (Britton and Dodd (1976)), and the conversion factor from energy flux to quantum flux in the PPF region of sunlight 4.57 µmol J^−1^ [49]. Greenhouse transmissivity (%) was calculated as:

Greenhouse transmissivity (%) = PPFD at plant level/(Global radiation × 0.47 × 4.57) × 100(1)

Where PPFD: photosynthetic photon flux density (µmol∙m−2∙s−1)

Global radiation outside the greenhouse was measured every five minutes. When the shading screen was closed, greenhouse transmissivity decreased by ~42%. To calculate PPFD supplied from supplemental light (SL), the number of hours during which SL was switched on was multiplied by its intensity. Light quality of a HPS and LED lamp was measured at 1 m distance with a field spectroradiometer (SS-110, Apogee instruments, Logan, UT, United States). Light intensity at plant level was measured with a quantum sensor (LI-COR, LI-190R Quantum Sensor).

### 4.3. Stomatal Conductance (g_s_) and Transpiration Rate (E)

All physiological measurements took place in weeks 3–4 after the start of treatments. The outermost three leaflets of the most fully expanded leaf were used (third or fourth leaf, depending on cultivar, counting from the base) per plant and three plants per genotype were measured. In all experiments, *g_s_* was measured with a porometer (Decagon Devices Inc., Pullman, WA, USA), which was calibrated daily with fresh desiccant to ensure accurate measurements. To confirm the results obtained with porometer and to measure *g_s_* and E at the same time, during experiments 3 and 4, the LI-6400 photosynthesis system (Li-Cor) was used. Using a transparent cuvette (enclosed leaf area: 6 cm^2^), leaflets were enclosed at 400 ± 1.3 µbar CO_2_, 22.4 ± 0.2 °C cuvette temperature, 70 ± 1% RH and a flow rate of 500 µmol s^−1^. After they had reached stability, *g_s_* and *E* were logged 10 times at 30 s intervals (to be sure we logged a representative value); these values were later averaged over the 3 biological replicates to increase accuracy. To minimize the effects of diurnal changes in temperature and light intensity, measurements of *g_s_* and *E* were performed alternatively between the HPS and LED compartment (three plants per compartment were alternatively measured).

### 4.4. Photosynthetic CO_2_ Response Curves

The response of net photosynthesis rate (A; µmol m^−2^s^−1^) to leaf internal CO_2_ partial pressure (C_i_, µbar) was determined during experiment 1, 3 and 4. The LI-6400, equipped with the 6400-40 fluorescence cuvette (90% R/10% B spectrum at peak intensities of 635 and 465 nm, respectively; enclosed leaf area: 2 cm^2^), was used. The measurements were done on one leaf per plant for three plants per genotype for each bench. Leaves were enclosed in the cuvette at 1500 µmol m^−2^s^−1^ PPFD, 101.5 ± 0.05 kPa atmospheric pressure, 23 ± 0.2 °C cuvette temperature, 70 ± 5% RH, a flow rate of 400 µmol air s^−1^ and 400 ± 2 µbar CO_2_ partial pressure. After waiting for A to stabilize (∼15 min), CO_2_ partial pressure was changed in steps to 300, 100, 50, 400, 400, 600, 850, 1100 and 1400 µbar, while all other environmental variables were kept constant. At each CO_2_ step (2–3 min duration), after A was stabilized, values of CO_2_ and H_2_O measured by the infrared gas sensor of the sample cell were calibrated against those of the reference cell (“matching”), and A was logged. A and C_i_ values were corrected for leaks of CO_2_ into or out of the cuvette, according to Long and Bernacchi [50]. From CO_2_ response curves, V_c,max_ (maximum carboxylation rate), J_1500_ (electron transport rate at 1500 μmol m^−2^ s^−1^) and TPU (maximum triose phosphate utilization rate) were determined according to Sharkey [51], including a leaf temperature correction to 25 °C. For fitting of CO_2_ response curves, mesophyll conductance was assumed to be 0.189 mol m^−2^s^−1^, as determined for young tomato leaves [52], while data from A vs. PPFD in the range 0–300 µmol m^−2^ s^−1^ PPFD (determined during experiment 1) were used to calculate day respiration (R_d_). A negative-exponential function was fitted to A/PPFD, and the resulting intercept value was used as R_d_. Since R_d_ was not significantly different between treatments (*p* = 0.14) or genotypes (*p* = 0.23), a global value of R_d_ = 2.1 ± 0.1 µmol m^-2^s^−1^ was used for fitting CO_2_ response curves.

### 4.5. Stomatal Density

In experiments 3 and 4, on 20 DAT, one stomatal imprint per biological replicate (with three biological replicates per genotype) was taken on the leaf abaxial side using the silicon rubber impression technique [53]. Stomatal density was measured on images (800-fold magnification) of the imprints (Figure S1) using a microscope (Leica, Aristoplan, Wetzlar, Germany) connected to a digital camera (DXM-1200, Nikon, Tokyo, Japan). Per imprint, stomata were counted on a randomly selected area of 0.25 mm^2^. Image processing and count of stomata was done using ImageJ (University of Texas Health Science Centre at San Antonio, TX, USA).

### 4.6. Leaf Temperature

Leaf temperature was measured with (i) an infrared non-contact thermometer (Raynger ST, Raytek, Santa Cruz, CA, USA) in experiment 3, and (ii) using a thermal camera (FLIR A655sc w/25° lens, 640 × 480, FLIR Systems, Ltd. Burlington, ON, Canada) in all four experiments. Measurements were conducted in weeks 3–4 after transplanting. For (i) and (ii), the temperature of the outermost three leaflets of the most fully expanded leaf, which was approximately perpendicular to the lamps, was measured. Measurements with the infrared thermometer took place one hour before sunrise, while lamps were switched on, to avoid the influence of solar radiation on leaf temperature. To correct for the different temperatures at plant level in the HPS and LED compartments, the temperature of a reference leaf (green printer paper Gemini green, 90 g m^−2^, Astrobrights by Wausau papers, Mosinee, WI, USA) was also measured. 

Using the thermal camera, leaf temperature readings from thermal images were taken over a circle with an area of 6 cm^2^. Per measurement, 30 images were captured for 15 min (1 image was captured every 30 s) and an average temperature was calculated. Leaf heat emissivity was assumed to be 0.95 [46]. To minimize the effects of diurnal changes in air temperature on leaf temperature, images were recorded alternatively in HPS and LED compartments (three plants from different genotype per compartment were alternatively measured). Reference surfaces were used to establish a temperature range to simulate leaves at 100% (wet reference) and 0% (dry reference) transpiration. Reference surfaces were used to normalize the data obtained per measurement. Green printer paper (Gemini green) was used as dry reference and white filter paper grade 597 (VWR International LLC, Radnor, PA, USA), which was sprayed continuously with distilled water, was used as a wet reference. These materials were cut into shapes resembling tomato leaflets and attached to sticks, so the papers were hanging freely in the air.

### 4.7. Plant Water Stress Indices

From the thermal images, the thermal index (I_G_) [44] was calculated as:

I_G_ = (T_dry_ − T_leaf_)/(T_leaf_ − T_wet_); (2)

with T_dry_ as dry reference, T_leaf_ as leaf and T_wet_ as wet reference temperature, respectively. The value of I_G_ can vary from 0 (no transpiration) to ∞ (maximal transpiration); and the crop water stress index (CWSI) [45] was calculated as:

CWSI = (T_dry_ − T_leaf_)/(T_dry_ − T_wet_) (3)

CWSI can vary from 0 (no transpiration) to 1 (maximal transpiration).

### 4.8. Statistical Analysis

Each experiment was considered as an independent activity. Three replicate plants per genotype were evaluated per experiment. Data were first tested for homogeneity (Levene’s test) and for normal distribution of variances (Shapiro–Wilk test). The experimental factors were fixed in a two-way analysis of variance (ANOVA). To determine differences between means, a Tukey test was used. Correlations were tested using Pearson’s correlation coefficient. Analyses were performed using R (version 3.4.3, R Foundation for Statistical Computing, Vienna, Austria). Packages used for the analysis were: agricolae [54], multcompView [55], car [56] and ggpubr [57].

## 5. Conclusions

Leaves of young tomato plants under LED showed higher gas exchange and photosynthetic capacity compared to HPS; this may be related to the increased leaf temperature under HPS, causing subtle but detectable differences in environmental acclimation. The fact that leaves were warmer under HPS lamps could be a result of the higher heat radiation (NIR light) relative to LED lamps. Moreover, the presence of green light in HPS lamps may have led to stomatal closure and/or reduced stomatal density, in turn reducing transpiration and increasing leaf temperature. Genotypic differences were observed for *E*, *g_s_*, stomatal number and photosynthetic capacity. However, no genotypic differences were identified for leaf temperature. At higher ratios between natural light and SL (experiment 4), differences between HPS and LED were smaller. Finally, our results demonstrate that thermography is a potentially rapid and noninvasive technique to study plant water status and predict *g_s_*. However, further studies on, e.g., validation under a wide range of different conditions will be necessary to improve the accuracy of this prediction.

## Figures and Tables

**Table 1:** Stomatal conductance (measured using a porometer) in seven tomato genotypes grown under HPS and LED supplemental light, in four experiments differing in fraction of SL over natural light (treatments: *n* = 21; genotypes: *n* = 3). Significance was tested by two-way ANOVA. Means followed by different letters within a column indicate significant differences according to Fisher’s protected LSD test (*p =* 0.05). SL—supplemental light treatments; Gn—genotypes**;** SL × Gn—interaction between SL and G_n_.

	Stomatal Conductance (mol H_2_O m^−2^ s^−1^)			
Experiment	1	2	3	4
Treatments (SL)				
HPS	0.32 b	0.37 b	0.59 b	0.56 b
LED	0.35 a	0.40 a	0.66 a	0.60 a
Genotypes (G_n_)				
Moneymaker	0.36 a	0.39 ab	0.62 b	0.56 cd
Momotaro	0.36 a	0.41 a	0.61 b	0.54 d
LA1578	-	0.36 b	0.73 a	0.66 a
Rutgers	0.31 b	0.36 b	0.56 c	0.50 e
Kentucky Beefsteak	0.29 b	0.38 ab	0.63 b	0.62 b
Nunhems-FM001	0.35 a	0.41 a	0.60 bc	0.62 b
Ailsa Craig	0.32 b	0.40 a	0.61 b	0.58 c
Significance ^1^				
SL	*	*	**	***
G_n_	***	*	***	***
SL × G_n_	*	***	NS	**

^1^ Significance: ***, **, and * for *p* ≤ 0.001, *p* ≤ 0.01, and *p* ≤ 0.05, respectively; NS—not significant.

**Table 2:** Stomatal density ± SE (mm^−2^) in seven tomato genotypes grown under HPS and LED supplemental light, in two experiments differing in fraction of SL over natural light (treatments: *n* = 21; genotypes: *n* = 3). Significance was tested by two-way ANOVA. Means followed by different letters within a column indicate significant differences according to Fisher’s protected LSD test (*p = 0.05*). SL—supplemental light treatments; G_n_—genotypes**;** SL—G_n_—interaction between SL and Gn.

	Stomatal Density (Number mm^−2^)	
Experiment	1	4
Treatment (SL)		
HPS	185 ± 9 b	186 ± 7 b
LED	216 ± 11 a	205 ± 8 a
Genotype (G_n_)		
Moneymaker	192 ± 13 bcd	176 ± 6 c
Momotaro	195 ± 13 bc	188 ± 13 bc
LA1578	276 ± 14 a	347 ± 7 a
Rutgers	168 ± 8 cd	135 ± 8 d
Kentucky Beefsteak	214 ± 11 bc	193 ± 7 bc
Nunhems-FM001	216 ± 11 b	210 ± 10 b
Ailsa Craig	95 ± 11 d	136 ± 7 d
Significance ^1^		
*SL*	***	**
G_n_	***	***
SL × G_n_	NS	NS

^1^ Significance: *** and ** for *p* ≤ 0.001 and *p* ≤ 0.01, respectively; NS—not significant.

**Table 3:** Leaf temperature ± SE (°C) in seven tomato genotypes grown under HPS and LED supplemental light, in four experiments differing in fraction of SL over natural light (treatments: *n* = 21; genotypes: *n* = 3). Significance was tested by two-way ANOVA. Means followed by different letters within a column indicate significant differences according to Fisher’s protected LSD test (*p =* 0.05). SL—supplemental light treatments.

	Leaf Temperature (°C)			
Experiment	1	2	3	4
Treatment (SL)				
HPS	23.8 ± 1.4 a	21.9 ± 1.0 a	22.0 ± 1.5 a	23.4 ± 0.9 a
LED	22.0 ± 1.2 b	21.1 ± 0.7 b	22.4 ± 1.0 a	23.1 ± 1.2 a
Significance ^1^				
SL	***	***	NS	NS

^1^ Significance: *** for *p* ≤ 0.001; NS—not significant.

**Table 4:** Crop water stress index (CWSI) in seven tomato genotypes grown under HPS and LED supplemental light, in four experiments differing in fraction of SL over natural light (treatments: *n* = 21; genotypes: *n* = 3). Significance was tested by two-way ANOVA. Means followed by different letters within a column indicate significant differences according to Fisher’s protected LSD test (*p = 0.05*). SL—supplemental light treatments; G_n_—genotypes**;** SL × G_n_—interaction between SL and Gn.

	Crop Water Stress Index (CWSI)			
Experiment	1	2	3	4
Treatments (SL)				
HPS	0.53 b	0.54 b	0.65 b	0.68 a
LED	0.58 a	0.66 a	0.69 a	0.69 a
Genotypes (G_n_)				
Moneymaker	0.63 a	0.70 a	0.63 de	0.70 b
Momotaro	0.56 b	0.63 b	0.70 b	0.64 de
LA1578	-	0.62 b	0.69 bc	0.76 a
Rutgers	0.58 ab	0.61 b	0.59 e	0.61 e
Kentucky Beefsteak	0.46 c	0.59 b	0.67 bcd	0.67 bc
Nunhems-FM001	0.50 c	0.58 b	0.65 cd	0.66 cd
Ailsa Craig	0.59 ab	0.48 c	0.75 a	0.75 a
Significance ^1^				
*SL*	**	***	**	*NS*
G_n_	***	***	***	***
SL × G_n_	*NS*	***	***	*NS*

^1^ Significance: *** and ** for *p* ≤ 0.001 and *p* ≤ 0.01, respectively; NS—not significant.

**Table 5:** Spectral characteristics of LED and HPS fixtures. For blue, green and red, spectral composition (%) was calculated relative to the PPF range (400–800 nm) and rounded to an accuracy of 5%.

Light Quality Parameters	Supplemental Light	
	LED	HPS
% Blue (400–500 nm)	5	5
% Green–Yellow (501–600 nm)	0	50
% Red (601–700 nm)	95	40
% Far–Red (701–800 nm)	0	5
(Green–Yellow):Blue	0	10
Red:Far Red	NA	965

## Data Availability

The raw data supporting the conclusions of this article will be made available by the authors, without undue reservation.

## Supplementary Materials

The following are available online at https://www.mdpi.com/article/10.3390/plants10040810/s1, Figure S1: Stomatal imprint of cv. ‘Rutgers’ grown under LEDs supplemental light; Figure S2: Daily light integral from natural light (DLI_nl_), from supplemental light (DLI_sl_) and the sum of DLI from supplemental light plus natural light (DLI_n+sl_); Figure S3: Air temperature (°C) in greenhouse compartments; Table S1: Stomatal conductance (*g_s_*) measured with the porometer per genotype and experiment.; Table S2: Average stomatal conductance and transpiration rate (measured with LI-6400 XT) in seven tomato genotypes grown under HPS and LED supplemental light; Table S3: Thermal Index (Ig) per treatment (*n* = 21) and genotype (*n* = 3). Table S3: Thermal Index (I_g_) per treatment (*n* = 21) and genotype (*n* = 3).

## Abbreviations

*A* (net photosynthesis rate; μmol m^−2^ s^−1^); B (blue light); CWSI (crop water stress index); DAT (days after start of treatments); DLI (daily light integral; mol m^−2^ d^−1^); *E* (transpiration rate; mol H_2_O m^−2^ s^−1^); FR (far-red light); G (green light); G_n_ (genotype); *g_s_* (stomatal conductance; mol H_2_O m^−2^ s^−1^); HPS (high pressure sodium); I_G_ (thermal index); IR (infrared); J_1500_ (maximum electron transport rate at 1500 μmol m^−2^ s^−1^; μmol m^−2^ s^−1^); LED (light emitting diodes); PPF (photosynthetic photon flux; μmol m^−2^); PPFD (photosynthetic photon flux density; μmol m^−2^ s^−1^); R (red light); R_d_ (day respiration; µmol m^−2^s^−1^); RH (relative humidity; %); SL (supplemental light); T_dry_ (dry reference temperature; °C); T_leaf_ (leaf temperature; °C); TPU (maximum triose phosphate utilization rate; μmol m^−2^ s^−1^); T_wet_ (wet reference temperature; °C); V_c,max_ (maximum carboxylation rate; μmol m^−2^ s^−1^); Y (yellow light).
