# Supplementary material for: LED and HPS Supplementary Light Differentially Affect Gas Exchange in Tomato Leaves
Source: Plants (Basel). 2021 Apr 20;10(4):810. doi: 10.3390/plants10040810 (PMC8074298; doi:10.3390/plants10040810)
Supplement: Supplementary file 1 [file plants-10-00810-s001.zip › plants-1162526-supplementary.pdf]

Supplementary Material

Supplementary Tables

Table 1. Stomatal conductance (g_s) measured with the porometer per genotype and experiment. The average g_s (n = 9) as well as the ratio of gs in LED to HPS light is displayed. A significance of the difference between the light treatment groups HPS and LED is shown according to the p-value of a t-test: * p-value <0.05, ** p-value < 0.01, *** p-value < 0.001.

Genotype	Exp.	Stomatal conductance (gs) in HPS	Stomatal conductance (gs) in LED	ratio LED/HPS	*Significance*
	(I-IV)	(mol H_2O m^{-2} s^{-1})			
Ailsa Craig	I	271.1	360.9	1.33	***
Ailsa Craig	II	352.2	446	1.27	**
Ailsa Craig	III	571.4	658.5	1.15	***
Ailsa Craig	IV	560	595.6	1.06	.
Kentucky Beefsteack	I	280.6	298.6	1.06	.
Kentucky Beefsteack	II	353.9	411.9	1.16	.
Kentucky Beefsteack	III	587.6	669.6	1.14	**
Kentucky Beefsteack	IV	592	640.5	1.08	.
LA1578	II	395.6	326.1	0.82	.
LA1579	III	709.3	758.5	1.07	.
LA1580	IV	675.5	637.7	0.94	.
Momotaro	I	356.7	354.6	0.99	.
Momotaro	II	422.3	360.3	0.85	**
Momotaro	III	549.6	694.2	1.26	***
Momotaro	IV	506.2	605	1.2	*
Moneymaker	I	369	349.7	0.95	.
Moneymaker	II	372.9	449.2	1.2	*
Moneymaker	III	572.8	645.6	1.13	**
Moneymaker	IV	490.7	581.2	1.18	.
Nunhems-FM001	I	393	375.3	0.95	.
Nunhems-FM002	II	388.8	425.9	1.1	.
Nunhems-FM003	III	575.7	616.1	1.07	.
Nunhems-FM004	IV	606.7	631.2	1.04	.
Rutgers	I	277.5	354.9	1.28	**
Rutgers	II	339.2	375.3	1.11	.
Rutgers	III	546.8	574.6	1.05	.
Rutgers	IV	463.5	531.8	1.15	.

Table 2. Average stomatal conductance and transpiration rate (measured with LI-6400 XT) in seven tomato genotypes grown under HPS and LED supplemental light, during the third and fourth Expt. (n = 21) and different genotypes (n = 3). An ANOVA was done to determine the overall effect of the treatment or genotypes on the stomatal density. Significance: ***, **, and * for $p \leq 0.001$, $p \leq 0.01$, and $p \leq 0.05$, respectively; NS—not significant. Per genotype and between the treatments, a post hoc test was performed and the significant different groups are indicated by letter. Identical letters in the same column indicate that means are not significantly different (p > 0.05).

	Stomatal conductance (molH_2O $m^{-2}s^{-1}$)		Transpiration rate (mmolH_2O $m^{-2}s^{-1}$)	
	Expt. 3	Expt. 4	Expt. 3	Expt. 4
**Treatments** (SL)				
HPS	0.61 b	0.77 b	3.12 b	5.46 b
LED	0.74 a	0.87 a	4.13 a	5.57 a
**Genotypes** (G)				
Moneymaker	0.70 b	0.82 d	3.81 b	5.52 d

Momotaro	0.74 a	0.76 f	3.90 a	5.36 f
LA1578	0.67 c	0.95 a	3.62 d	5.91 a
Rutgers	0.52 d	0.72 g	3.27 g	5.20 g
Kentucky Beefsteack	0.67 c	0.79 e	3.49 f	5.45 e
Nunhems-FM001	0.69 b	0.83 c	3.51 e	5.53 c
Ailsa Craig	0.75 a	0.85 b	3.78 c	5.63 b
Significance				
SL	***	***	***	***
G	***	***	***	***
SL*G	***	**	***	**

Table 3. Thermal Index (Ig) per treatment (*n* = 21) and genotype (*n* = 3). Within the same main effect and for each parameter, the same lowercase letters in the same column indicate that the mean values are not significantly different ($p = 0.05$). An ANOVA was done to determine the overall effect of the treatment or genotypes on the stomatal density. Significance: ***, **, and * for $p \leq 0.001$, $p \leq 0.01$, and $p \leq 0.05$, respectively; NS—not significant. Per genotype and treatment, a post hoc test was performed and the significant different groups are indicated by letter. Identical letters in the same column indicate that means are not significantly different ($p > 0.05$).

	**Thermal Index (Ig)**			
	**Expt. 1**	**Expt. 2**	Expt. 3	Expt. 4
**Treatments** (SL)				
HPS	1.2 b	1.4 b	2.1 b	2.3 a
LED	1.5 a	2.1 a	2.5 a	2.4 a
**Genotypes** (G)				
Moneymaker	1.8 a	2.5 a	1.9 cd	2.4 b
Momotaro	1.3 bc	1.8 b	2.5 b	1.8 cd
LA1578	-	1.8 bc	2.4 b	3.3 a
Rutgers	1.5 ab	1.7 bc	1.6 d	1.7 d
Kentucky Beefsteack	0.9 d	1.5 cd	2.4 b	2.1 bc
Nunhems-FM001	1.0 cd	1.5 bcd	2.2 bc	1.9 cd
Ailsa Craig	1.7 a	1.3 d	3.1 a	3.1 a
Significance				
SL	**	***	**	*NS*
G	***	***	***	***
SL*G	*NS*	***	***	*NS*

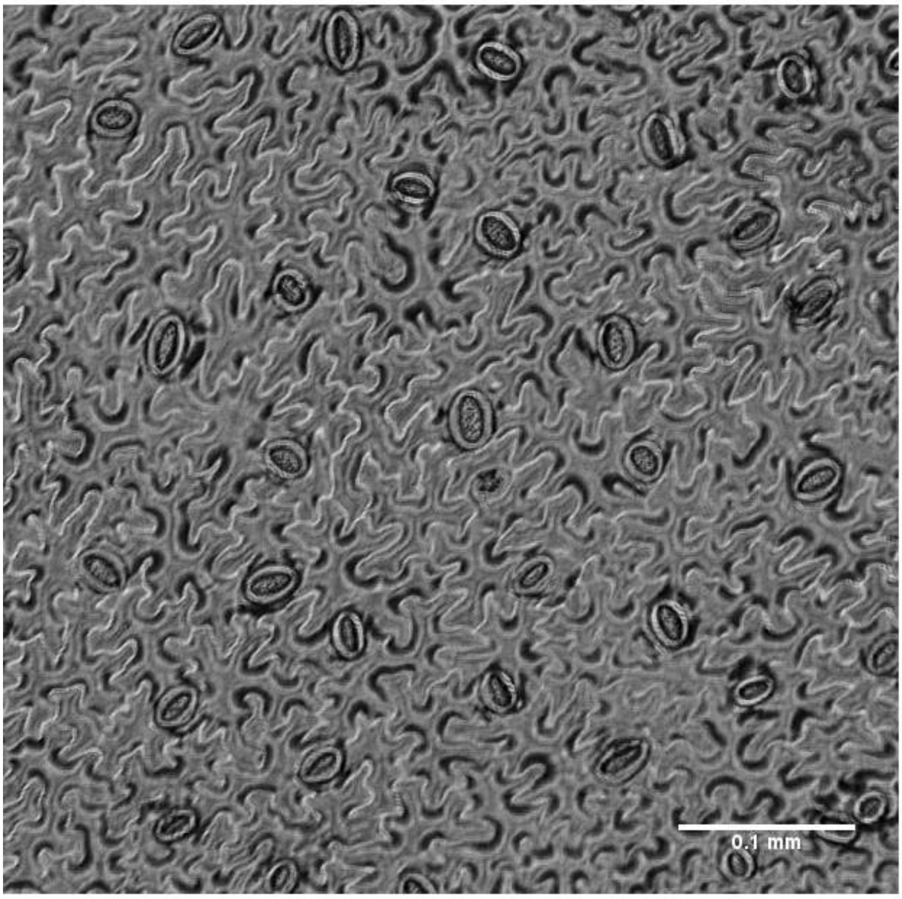

Figure 1. Stomatal imprint of cv. 'Rutgers' grown under LEDs supplemental light during the fourth trial.

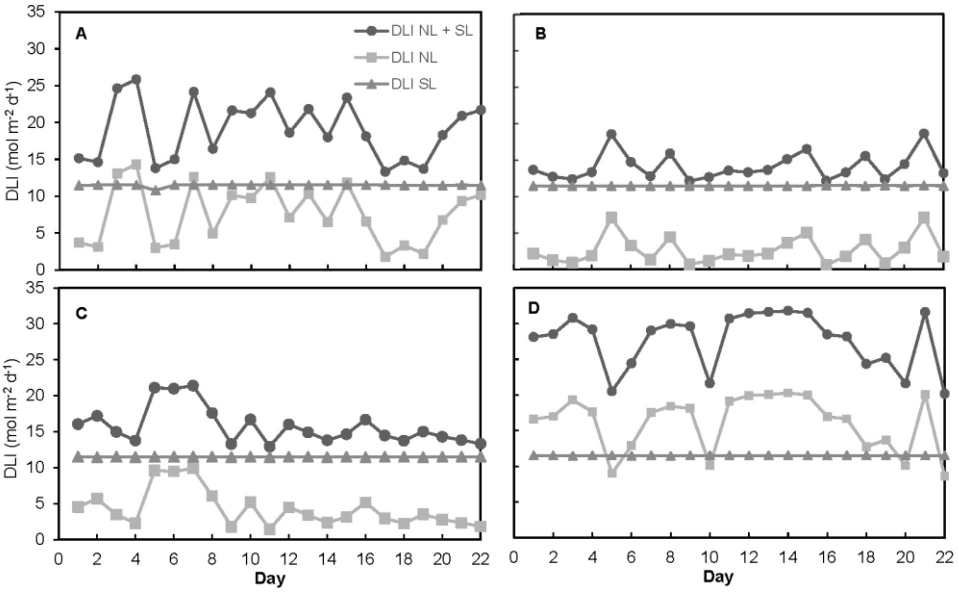

Figure 2. Daily light integral from natural light (DLI NL), from supplemental light (DLI SL) and the sum of DLI from supplemental light plus natural light (DLI NL + SL), in both treatment conditions, during experiments 1–4 (**A**–**D**).

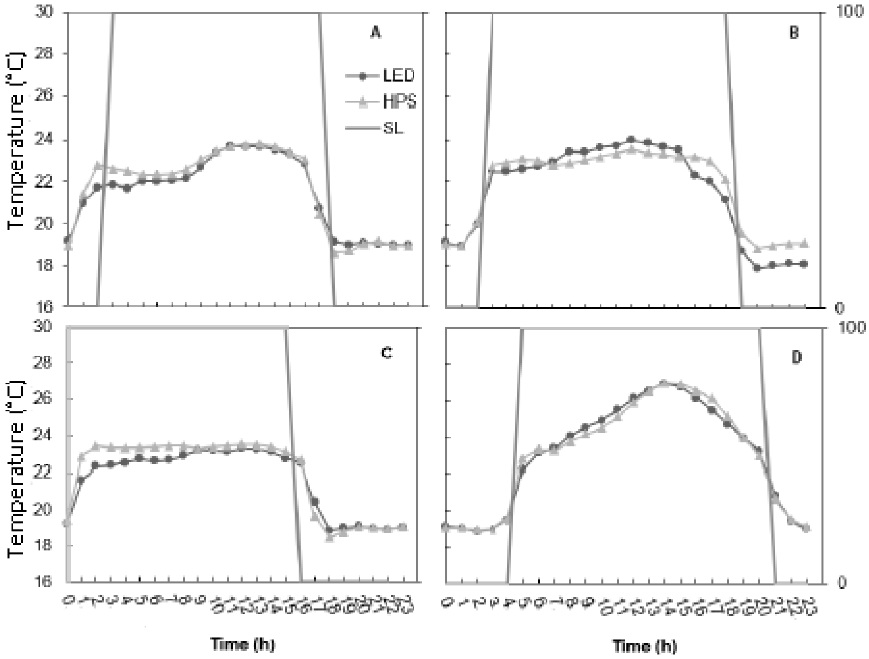

Figure 3. Air temperature (in °C) in greenhouse compartments provided with HPS and LEDs supplemental light during Table 1. (**A**), Expt. 2 (**B**), Expt. 3 (**C**) and Expt. 4 (**D**). Each point is the average value of 6 measurements per hour for 22 days.
